# Supplementary material for: Exercise-Induced ADAR2 Protects against Nonalcoholic Fatty Liver Disease through miR-34a
Source: Nutrients. 2022 Dec 27;15(1):121. doi: 10.3390/nu15010121 (PMC9824461; doi:10.3390/nu15010121)
Supplement: Supplementary file 1 [file nutrients-15-00121-s001.zip › nutrients-2064211-supplementary.pdf]

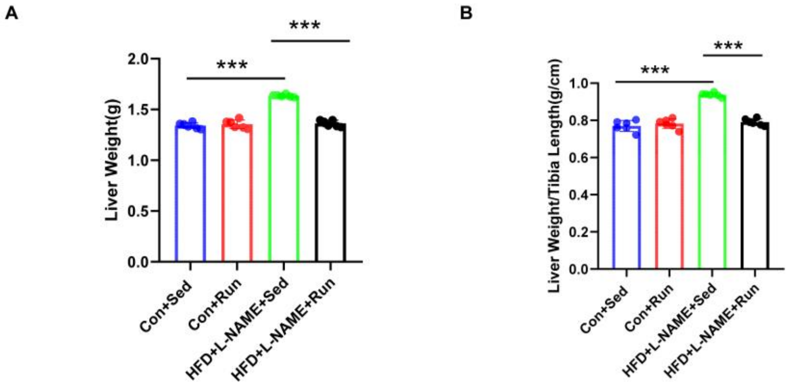

Figure S1. The liver weight of NAFLD/control mice after exercise or sedentary for 12 weeks. (A) The liver weight of mice. (B) The liver weight / tibia length of mice. ***P < 0.001; n=6. Sed, sedentary ; HFD, high-fat diet; L-NAME, Nω-nitro-L-arginine methyl ester, hydrochloride.

Table S1 The sequences of primers used for RT-PCR

Gene	Primer sequence (5'-3')
mmu-*Gapdh*-Forward	TGTGTCCGTCGTGGATCTGA
mmu-*Gapdh*-Reverse	CCTGCTTCACCACCTTCTTGAT
mmu-*Adar2*-Forward	GTATGACGCCAGACTCTCACCA
mmu-*Adar2*-Reverse	CAGGTCTGGATGCTGGCATTTG
mmu-*Fasn*-Forward	CACAGTGCTCAAAGGACATGCC
mmu-*Fasn*-Reverse	CACCAGGTGTAGTGCCTTCCTC
mmu-*Srebp1c*-Forward	CGACTACATCCGCTTCTTGCAG
mmu-*Srebp1c*-Reverse	CCTCCATAGACACATCTGTGCC
hsa-*Gapdh*-Forward	GTCTCCTCTGACTTCAACAGCG
hsa-*Gapdh*-Reverse	ACCACCCTGTTGCTGTAGCCAA
hsa-*Fasn*-Forward	TTCTACGGCTCCACGCTCTTCC
hsa-*Fasn*-Reverse	GAAGAGTCTTCGTCAGCCAGGA
hsa-*Srebp1c*-Forward	ACTTCTGGAGGCATCGCAAGCA
hsa-*Srebp1c*-Reverse	AGGTTCCAGAGGAGGCTACAAG
